# Cheilitis glandularis: Case report with respect to immunohistochemical findings

**DOI:** 10.3205/iprs000083

**Published:** 2016-01-20

**Authors:** Reinhard E. Friedrich, Thomas Löning

**Affiliations:** 1 Oral and Craniomaxillofacial Surgery, Eppendorf University Hospital, University of Hamburg, Germany; 2 Gerhard-Seifert Reference Centre, Hansepathnet, Hamburg, Germany

**Keywords:** cheilitis glandularis, lip surgery, minor salivary gland, IgG4-related disease

## Abstract

Cheilitis glandularis (CG) is a rare benign affection of the lip mucosa. The etiology and pathogenesis of CG are unknown. Surgical measures are the leading therapeutic options to treat CG. This case report on a 55-year-old female illustrates the recurrent affection of the lips over several years and local therapy. Furthermore, the study of the resected glands intends to differentiate the lesions with respect to the recently introduced concept of immunoglobulin G4-related diseases.

## Zusammenfassung

Die Cheilitis glandularis ist eine seltene, gutartige Affektion der Lippenschleimhaut. Die Ätiopathogenese ist unbekannt. Chirurgische Maßnahmen sind in der Behandlung führend. Der Fallbericht einer 55-jährigen Frau beschreibt das wiederholte Auftreten von typischen Befunden über mehrere Jahre und die lokale Therapie. Darüber hinaus unternimmt die Untersuchung der Exzidate den Versuch, die Läsionen in Zusammenhang mit den neuerlich eingeführten IgG4-assoziierten Erkrankungen zu differenzieren.

## Introduction

Cheilitis glandularis (CG) is a rare chronic inflammatory condition of the minor salivary glands, predominantly of the lower lip [1]. CG usually affects the vermilion of the lips, and several reports have addressed the frequent history of extended exposure to sunlight in the pathogenesis of CG [2], [3]. The disease was first described by von Volkmann under the designation ‘cheilitis glandularis apostematosa’ or ‘myxadenitis labialis’ [4], [5]. In addition to the preferred location of CG in minor salivary glands there are also some reports that provide evidence for other regions of the oral cavity giving rise to this benign lesion [6].

The aim of this report is to address the capacity of CG to develop local tumour recurrence and to present some peculiar histological features of the lesions.

## Case report

**Figure 1:**
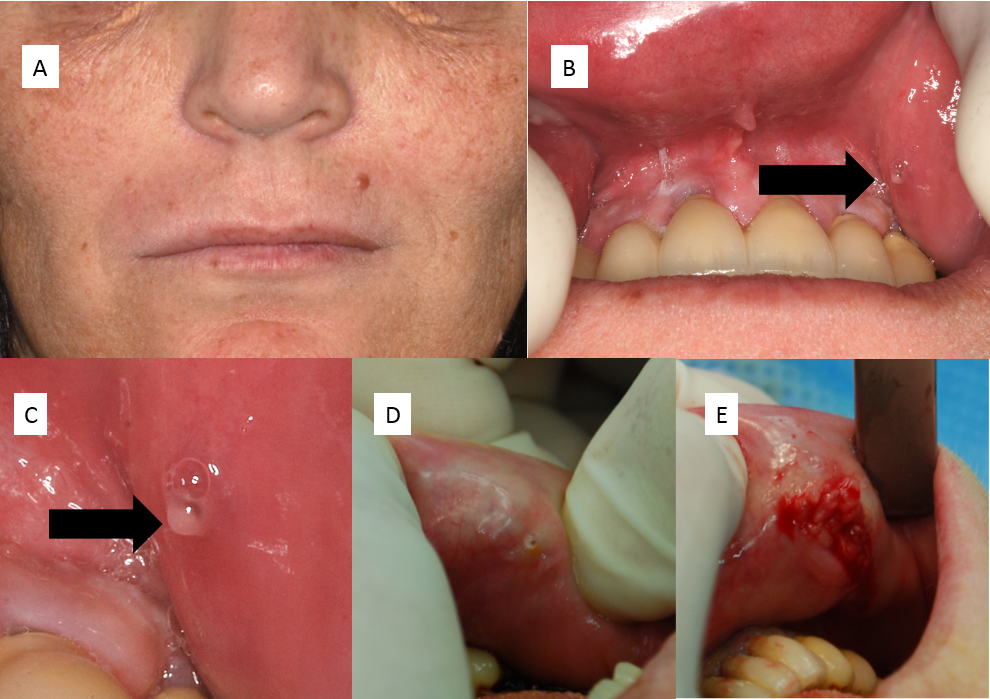
(A) Detail of patient’s photograph en face showing multiple pigmentary disorders of her light skin. The lips also show pigmentary disorders but the lip contour is not affected. The disease cannot be estimated from the external evaluation. (B) Oral inspection reveals clear mucous drooling after light compression of the upper lip (C) Close-up view of the oral aspect shown in (B) reveals the extended orifice of the labial minor salivary gland. (D) Expulsion of clear saliva after slight pressure on the lower lip from minor salivary glands of the lower lip vermilion during oral investigation for recurrent cheilitis glandularis. (E) After incision of the lower lip, berry-like enlarged minor salivary glands come to light.

The 55-year-old female patient attended the outpatient clinic of the oral and craniomaxillofacial surgery department for treatment of multiple painful nodules of her lip mucosa. On admission, the patient was in excellent general health and had no history of further diseases. She reported to have noticed an unspecific pressure on her lips during the preceding years but could not detail the history of her symptoms or the palpability of her findings. She denied any history of viral affection of the oral region. External examination showed no alteration of the lips (Figure 1A (Fig. 1)). Oral examination revealed multiple nodules, painful on palpation, below the non-inflammatory mucosa of the upper and lower lip. Enlarged ostia were visible that secreted a clear tenacious mucus (Figures 1B (Fig. 1) and 1C (Fig. 1)).

### Therapy

Local excision of the palpable masses was performed in all four quadrants of the vermilion. After incision, berry-like tumours were visible that lay beneath the mucous membrane and did not infiltrate the lip muscles (Figure 1E (Fig. 1)). Healing was uneventful after surgical removal of the well-delineated lesions. However, the patient returned four and five years after the first procedure to get relief from recurrent painful minor salivary gland swellings at the same site. The physical appearance of the lesion was always the same throughout the observation period of almost six years (Figure 1D (Fig. 1)).

### Histology

**Figure 2:**
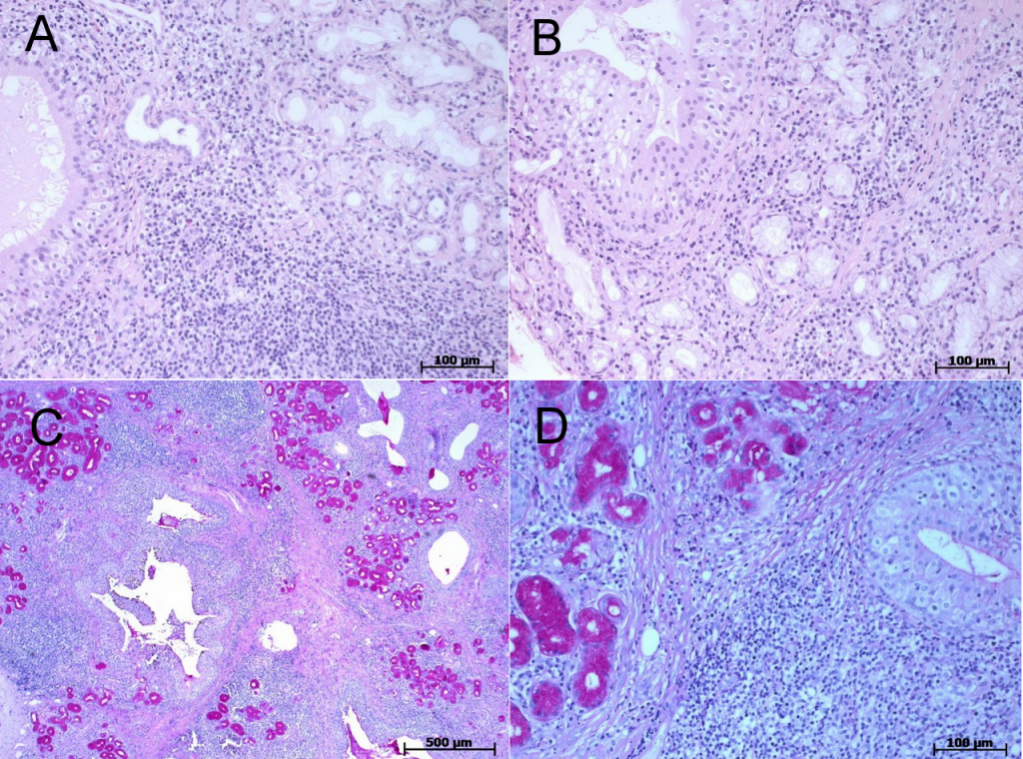
(A) Minor salivary gland with interstitial lymphoid infiltrates penetrating atrophic acinar residues; excretory ducts (left) showing moderate ectasia and mucous retention. (B) Closer look at the duct system showing epithelial hyperplasia and apocrine metaplasia (A and B: haematoxylin-eosin staining). (C) Low magnification of the ectatic ductal tree and the irregular atrophia of acinar tissues with some fibrosis and lymphoid infiltrates in between. (D) Closer look at the lymphoid infiltrates penetrating the hyperplastic ductal epithelium (C and D: periodic acid-Schiff reaction).

 The specimen showed multiple ductal ectasia of minor salivary glands and oncocyte metaplasia. Inflammatory reaction of the interstitium was seen and the acini showed irregular atrophia. Initially, a mechanical outflow obstruction was suggested as the cause of salivary gland enlargement, possibly due to trauma or malformation. Following local recurrence of the glandular swelling and renewed operation, a generalised chronic lymphoid sialadenitis was diagnosed with focal acute sialectasia. There were neither eosinophil granulocytes, granuloma nor cellular atypia (Figure 2 (Fig. 2)).

### Immunohistochemistry

**Figure 3:**
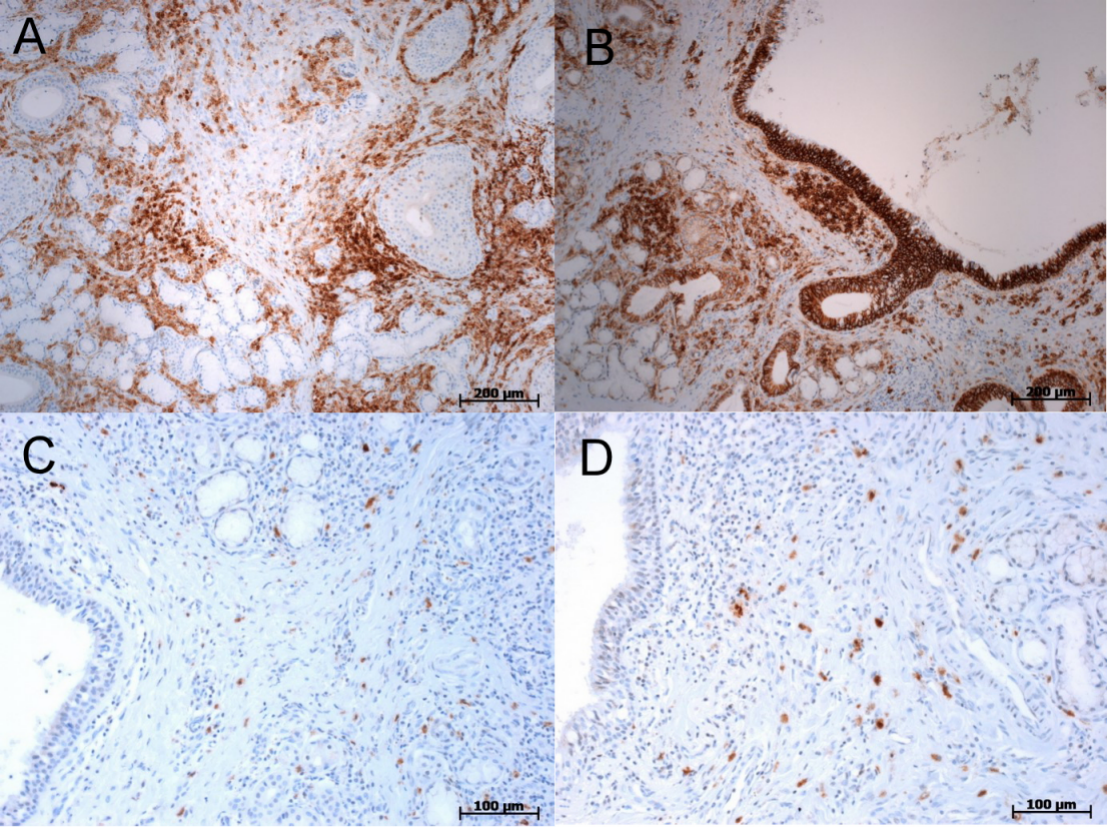
Immunohistochemical decoration of the interstitial lymphoid infiltrate (A: CD45, B: CD138). IgG4 staining showing a scattered pattern in (C), and very focal clustering in (D).

Stainings were done for CD45 (Figure 3A (Fig. 3)), CD138 (Figure 3B (Fig. 3)), and IgG4 (Figures 3C (Fig. 3) and 3D (Fig. 3)), and revealed lymphoplasmacellular infiltrates (see Figure 3A (Fig. 3)), mainly located in broadened interstitial spaces with some exocytosis in the ductal and acinar compartments. Immunoglobulin G subclass 4 (IgG4)-positive cells were mostly scattered, yet also showed some clustering (Figure 3B (Fig. 3)). Nevertheless, when referring to the total (CD138-positive) plasma cell population, IgG4-positive cells accounted for 5% at the utmost.

### Laboratory

Antinuclear antibody (ANA) screening (human epithelial type 2 (HEP 2) cells) revealed a slightly evaluated titre (1:160; reference: 1:80) and a microscopically defined finely speckled reaction pattern, typically found in, e.g. SSA/Ro. Furthermore, an unknown cytoplasmic fluorescence pattern was noted. The level of IgG4 was very low: 0.01 g/L (reference: 0.052–1.25 g/L). C-reactive protein was intermittently elevated (maximum 62 mg/L). Quantitative assessment of other parameters revealed normal values (ds-DNA-, U1 RNP-, Sm-, SSA-, SSB-, Scl-70-, centromere- and Jo-1-antibodies).

## Discussion

This report details the diagnosis and therapy of a patient affected with CG who needed repeated surgical treatment for recurrent disease. Furthermore, an attempt has been made to clarify potential association of this localised but multiple salivary gland-affecting disease with IgG4-related disorders. With respect to the immunohistochemical detection of IgG4+ plasma cells in the lesion, the reported case might even be discussed as a variant of IgG4-related diseases.

However, CG in the presented patient is somewhat different from the accepted phenotype: CG was reported to affect preferentially the lower lip of males. CG of females and children was only rarely reported [7], [8], [9].

### Diagnostic criteria

Reiter et al. [10] provided a review of 77 cases of CG published between 1950 and 2010 in order to establish diagnostic criteria for CG. According to these authors, two clinical criteria are mandatory in CG diagnosis: involvement of more than one minor salivary gland (multilocularity) and mucoid and/or purulent discharge (suppuration) from the apertures of the involved minor salivary glands. Furthermore, two or more histological criteria are also mandatory to speak of CG: sialectasia, chronic inflammation, mucous/oncocytic metaplasia (ducts and/or acini) and the presence of mucin in salivary ducts. With reference to this publication, the present case fulfils the currently proposed diagnostic criteria of CG [10].

### Etiology

The etiology of CG is unknown. Heredity was repeatedly brought into the discussion [5] with reference to an autosomal dominant trait [9], [11]. However, predisposing factors such as poor oral hygiene [9], [11], [12], smoking [13], [14], [15] and/or a compromised immune system [16], [17], [18] were also discussed as predisposing factors.

### Clinical appearance

CG has been classified into three types: simple, superficial suppurative, and deep suppurative. The original term *cheilitis glandularis apostematosa *[4], [15] is reserved for the deep, suppurative type of CG [18] and the superficial suppurative type is also known as Baelz disease [5]. Nowadays, these types are considered different stages belonging to the clinical spectrum of progression characteristics of CG [5].

### Squamous cell carcinoma (SCC)

Patients with CG are categorised as being at risk for the development of SCC of the lips [8]. The cause of this association was seen in the increased surface area and exposure to sunlight of the affected lip [8]. However, other reports give more weight to the context of genetically determined skin types and the association of CG. In this view on lip cancer pathogenesis, CG is an intermittent stage of sunlight exposure of skin with increased photosensitivity [3].

### Differential diagnosis

The clinical differential diagnosis of CG has to consider orofacial granulomatosis, multiple mucoceles and cystic fibrosis. However, clinical and morphological findings are quite characteristic for delineating CG from these entities [5]. Insufficient incisional biopsy may direct the suspected diagnosis in the wrong direction, in particular in cases with unusual histopathologic features imitating neoplastic lesions [17]. However, histological investigation of the specimen after full excision of the lesion will disclose the reactive process [17]. Occasionally, cheilitis granulomatosa may pose diagnostic problems when seen with monosymptomatic Melkersson-Rosenthal syndrome [8]. However, granulomas are not a feature of CG. On the other hand, Küttner’s tumour of the lip may imitate some features of CG in very rare instances. However, in this case, no mucous discharge of minor salivary glands in the tumour region was noted, and clinical and histological differentiation to CG was not found [19], [20]. Nevertheless, Küttner’s tumour is currently associated with IgG4-related disease [21].

### Pharmacotherapy

Intralesional corticosteroid therapy was recommended [8] but usually the long-term application and consecutive side effects of this medication cannot be avoided. Furthermore, according to other reports this regime proved to be of little or no help [3].

### Surgery

As a definite treatment, it was repeatedly recommended to excise the affected mucosal labial surface followed by a skin graft insertion to cover the defect. A contoured graft allows adequate lip mobility. This therapeutic regime could result in a definite cure, but clinical studies detailing the long-term follow-up are presently lacking [3], [5], [8]. In the presented case, the patient rejected extended operations and wished explicitly for local excision of interfering nodules, being anxious of subclinical tumour regrowth. Local excision is an established method to give relief from CG [6]. Recurrence of CG after surgical removal is usually rare [22], [23], [24]. However, short-term follow-up control is insufficient to declare recurrence-free local findings [5].

CG can occur in association with systemic diseases, e.g. HIV infection [16]. However, this coincidence is very likely an extremely rare finding. On the other hand, a current concept ascribes some localised chronic salivary gland diseases to a pathological autoimmune response syndrome, i.e. the IgG4-related diseases [21].

### Immunoglobulin G4-related disease

Recently, IgG4-related diseases were defined by Umehara et al. [25]. As mandatory findings to establish diagnosis, they claimed both elevated serum IgG4 levels (>135 mg/dL) and the presence of IgG4-bearing plasma cells. According to these authors, diagnosis further affords the number of IgG4-producing cells exceeding 40% of total IgG-expressing plasma cells, and the presence of tissue fibrosis or even sclerosis [25]. However, more recently, another study group reported that a relevant subset of cases with a high percentage of IgG4-positive cells (20–30%) showed normal serum IgG4 levels [26]. This finding allows us to conclude that a normal level of IgG4 does not exclude IgG4-related disease [27]. Even the cut-off point, i.e. the critical percentage of tissue IgG4-positive cells, is still a matter of debate, with some authors regarding any clustering of IgG4-positive cells as suspicious.

Next to the pancreas (see under autoimmune pancreatitis), the head and neck, particularly the salivary glands, are the second most frequently affected regions [28]. Küttner’s tumour was addressed as a potential manifestation of IgG4-related disease [21]. However, in a recent study on IgG4 expression, no such association could be revealed. These authors identified IgG4-positive plasma cells in the glands, yet attributed this response to a non-specific chronic inflammatory reaction [29]. In order to better understand true Küttner cases, Furukawa et al. [30] sorted out patients presenting with sialoliths in specimens. Interestingly, only patients with Küttner tumours without sialoliths showed bilateral swelling (37.5%), strong infiltration of IgG4-positive plasma cells (87.5%) and a history of other IgG4-related disease in a subset of affected individuals (12.5%) [30]. Lip biopsy was seen to contribute to this diagnosis [31]. However, the reliability of lip biopsies in the differential diagnosis of IgG4-related diseases is controversially discussed [32], [33]. Clinicians should be aware that other sites in the oral cavity can develop IgG4-expressing tumours [34].

Overall, the combined research efforts of numerous investigator groups will explain our concepts of the pathogenesis of many diseases, hitherto defined as isolated entities, against a background of very similar immunological pathomechanisms [35], [36].

Given the poor knowledge about CG pathogenesis, better immunological markers are needed, analogous to those successfully used in Sjögren’s syndrome (non-informative in this case). As already outlined, IgG4 serum levels were only slightly elevated, and tissue IgG4 cell numbers (apart from some clustering) inconclusive to prove the relationship of the present case to an IgG4-related disorder.

## Conclusion

CG is a rare disease of the minor salivary glands with a remarkable disposition for local recurrence after local excision. The possibility of a systemic disease with local manifestations should always be considered in causal research. Initial findings suggest a relationship with IgG4-mediated autoimmune disorders.

## Notes

### Competing interests

The authors declare that they have no competing interests.
